# Obesity Is Independently Associated with Early Loss of Reduction After Casting in Pediatric Both-Bone Forearm Fractures: A Cohort Study of Children Aged 3–13 Years

**DOI:** 10.3390/medicina62030565

**Published:** 2026-03-18

**Authors:** Mehmet Yiğit Gökmen, Ahmet Yılmaz, Hasan Orkun Varmış, Özhan Pazarcı

**Affiliations:** 1 Department of Orthopaedics and Traumatology, Faculty of Medicine, Çanakkale Onsekiz Mart University, Çanakkale 17110, Türkiye; 2 Department of Orthopaedics and Traumatology, Adana City Training and Research Hospital, University of Health Sciences, Adana 01230, Türkiye; ahmetyilmaz-dr@hotmail.com (A.Y.); orkunvarmis@gmail.com (H.O.V.); dr.pazarci@gmail.com (Ö.P.)

**Keywords:** forearm injuries, radius fractures, ulna fractures, pediatric obesity, orthopedic procedures

## Abstract

*Background and Objectives*: Pediatric both-bone forearm fractures are commonly treated nonoperatively, yet early loss of reduction remains a clinically important problem. Childhood obesity may be associated with reduced early radiographic stability after closed reduction and casting. *Materials and Methods*: A retrospective single-center cohort study was performed, including children aged 3 to 13 years who presented between 2020 and 2023 with acute both-bone forearm fractures involving the radius and ulna. Patients were categorized as normal weight (BMI 5th–84th percentile) or obese (≥95th percentile); overweight children (85th–94th percentile) were excluded. Fracture morphology and level, initial management (operative vs. conservative), and early loss of reduction were recorded. Multivariable logistic regression was conducted within the conservative cohort to identify independent factors associated with loss of reduction. *Results*: A total of 895 patients were included (normal weight *n* = 633; obese *n* = 262). Obese children had a higher proportion of complete fractures than normal-weight children (82.4% vs. 61.9%, *p* < 0.001) and underwent operative management more frequently at index presentation (33.6% vs. 13.1%, *p* < 0.001). Among conservatively treated patients (*n* = 724), early loss of reduction occurred in 36 cases and was more common in obese than normal-weight children (13.2% vs. 2.4%, *p* < 0.001). In multivariable analysis, obesity was independently associated with loss of reduction (aOR 5.98; 95% CI 2.89–12.38; *p* < 0.001). *Conclusions*: In children aged 3 to 13 years with both-bone forearm fractures treated with casting, obesity was independently associated with early loss of reduction. Weight status may serve as a practical clinical risk marker to support counseling and closer early radiographic surveillance during nonoperative care.

## 1. Introduction

Forearm fractures are among the most common long-bone injuries in children, accounting for approximately 40% of all pediatric fractures [1]. While the majority of both-bone forearm fractures involving the radius and ulna in a single extremity can be successfully managed nonoperatively with closed reduction and casting, maintaining acceptable alignment until clinical union remains a persistent challenge, with redisplacement rates reported around 26% in conservative cohorts in contemporary syntheses [2]. Failure to maintain reduction can lead to malunion, persistent loss of forearm rotation, and the need for delayed corrective procedures, particularly when the fracture’s remodeling potential is exceeded [3].

Recent epidemiological data indicate that the combined prevalence of overweight and obesity among school-aged children commonly approaches 25–30% and continues to rise with age, underscoring the growing public health relevance of pediatric obesity and its potential clinical implications [4].

In recent years, the rising prevalence of childhood obesity has emerged as a distinct host factor influencing both injury patterns and treatment outcomes. Theoretically, excess adiposity creates a larger soft-tissue envelope that may hinder effective cast molding and compromise three-point fixation, which can be reflected by unfavorable cast indices in cases that subsequently fail [5,6]. Although several studies have suggested an association between high body mass index (BMI) and increased risk of redisplacement after casting, the current literature often suffers from conceptual inconsistencies. Specifically, some reports conflate primary management decisions (initial operative vs. conservative treatment) with post-reduction mechanical failure (loss of reduction after casting), which are distinct clinical endpoints that require separate analytical denominators [2,7]. Failure to distinguish between treatment selection bias and true mechanical instability after an initially acceptable reduction limits causal interpretation of the observed associations.

Furthermore, inclusion of near-skeletally mature adolescents introduces heterogeneity because remodeling capacity is inversely related to age and diminishes substantially as children approach physeal closure. In older children and adolescents, thresholds for acceptable angulation are commonly stricter than in younger children, complicating the definition of “treatment failure” in mixed-age cohorts [8]. If age-specific alignment criteria are not applied consistently, outcome misclassification may occur, particularly in studies combining early childhood and peri-adolescent patients within a single analytical model.

Therefore, there is a need for studies that use a stable research question and strictly separate treatment selection from subsequent conservative failure within a more homogeneous pediatric age group. By focusing on children aged 3 to 13 years, a cohort with substantial remodeling capacity and relatively uniform nonoperative treatment algorithms, it becomes possible to more accurately isolate the association between obesity and fracture stability. In addition, a clear operational definition of early loss of reduction based on predefined radiographic thresholds and a specified follow-up interval is essential to avoid ambiguity in outcome assessment.

The aim of this retrospective cohort study was to determine whether obesity is associated with early loss of reduction in children aged 3 to 13 years with both-bone forearm fractures managed conservatively. Early loss of reduction was defined a priori as secondary displacement exceeding age- and fracture-level-specific acceptable alignment thresholds within the initial follow-up period after an initially satisfactory closed reduction. In addition, we examined the relationship between weight status and initial management decisions, as well as fracture morphology and initial displacement severity, within this standardized pediatric population. By explicitly separating the denominator of all eligible fractures from the subset managed nonoperatively, the study was designed to distinguish between selection-related differences and true post-reduction mechanical failure.

## 2. Materials and Methods

### 2.1. Ethical Approval

Ethical approval for this study was obtained from the Clinical Research Ethics Committee of Adana City Training and Research Hospital (Meeting No: 142; Decision No: 3033; Date: 21 December 2023). The study was conducted in accordance with the principles of the Declaration of Helsinki and its subsequent amendments. Given the retrospective design and the use of anonymized clinical and radiographic data, the requirement for written informed consent was waived by the ethics committee.

### 2.2. Study Design, Setting, and Participants

This retrospective cohort study was conducted at the Department of Orthopaedics and Traumatology, Adana City Training and Research Hospital, Türkiye. The institutional electronic medical record system was screened to identify pediatric patients presenting between January 2020 and December 2023 with radiographically confirmed acute both-bone forearm fractures involving the radius and ulna. Patients were identified using ICD-10 diagnostic codes S52.4 and S52.6.

A total of 1240 patients were screened. Patients were excluded if they had open fractures, pathological or metabolic bone disease, polytrauma requiring immediate stabilization, Monteggia or Galeazzi fracture-dislocation patterns, or incomplete radiographic follow-up. After these exclusions, 1045 patients remained eligible for weight analysis.

**Figure 1:**
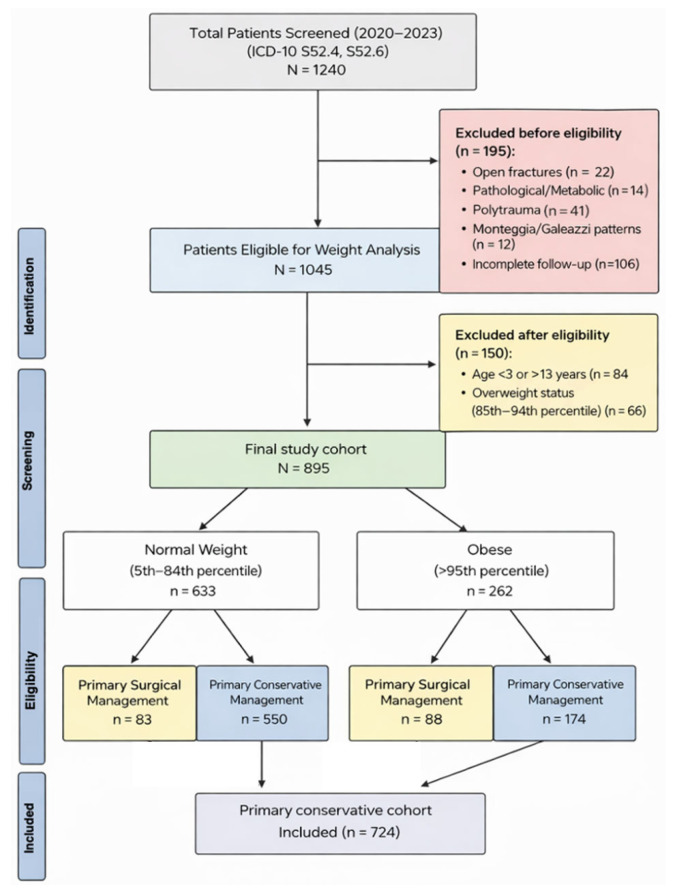
Flow diagram of patient selection, exclusion criteria, and cohort allocation for primary and conservative management analyses.

To create a homogeneous pediatric cohort with comparable remodeling potential and similar alignment thresholds, the analytic sample was restricted to children aged 3 to 13 years. Patients younger than 3 years or older than 13 years were excluded. To maintain a clear two-group comparison and reduce heterogeneity in weight classification, patients in the overweight range (85th to 94th BMI percentile) were excluded from the primary analysis. The final study cohort included 895 patients, categorized as normal weight (5th to 84th percentile; *n* = 633) and obese (95th percentile or higher; *n* = 262). The number and outcomes of excluded overweight patients were recorded separately to allow transparency regarding external validity. Patient selection and subgroup allocation are summarized in the study flow diagram (Figure 1).

Within the final cohort, treatment at initial presentation was categorized as either immediate surgical fixation or initial conservative management with closed reduction and casting, based on the treating surgeon’s clinical assessment and routine institutional practice. To avoid conflating treatment selection with post-reduction mechanical failure, all risk modeling for loss of reduction was strictly limited to the primary conservative cohort. The conservative cohort included 724 patients in total (normal weight *n* = 550; obese *n* = 174).

### 2.3. Anthropometric Assessment

Height and weight recorded at the index emergency department presentation were used to calculate body mass index as weight in kilograms divided by height in meters squared. BMI percentiles were determined using the CDC 2000 BMI-for-age growth charts [9]. Although CDC reference standards are based on the United States population, they are widely used in international pediatric research and provide standardized percentile-based categorization across age and sex strata. Patients were categorized into two predefined weight-status groups: normal weight (5th to 84th percentile) and obesity (95th percentile or higher). Patients with an overweight status (85th to 94th percentile) were excluded from the primary comparative analysis as described above.

### 2.4. Radiographic Evaluation and Fracture Characterization

All radiographs were retrieved from the institutional picture archiving and communication system. Standard anteroposterior and lateral forearm radiographs obtained at presentation, immediately after reduction when applicable, and during follow-up were reviewed by two orthopaedic surgeons experienced in pediatric trauma who were not involved in index treatment decisions.

Prior to data collection, both observers agreed on standardized operational definitions for fracture morphology, displacement severity, and measurement techniques. Fracture morphology was categorized as a complete fracture or a greenstick fracture based on the cortical disruption pattern. This variable was analyzed separately from displacement severity. Observers were blinded to patient weight status and treatment group allocation during radiographic assessment.

Fracture location was categorized according to the dominant level of injury as proximal third, mid-diaphyseal third, or distal third. When fractures involved different levels in the radius and ulna, classification was based on the more proximal fracture level, applied uniformly.

Radiographic measurements included angulation and translation on both projections. Angulation was defined as the angle between the longitudinal axes of the proximal and distal fragments at the fracture site. Translation was recorded as the percentage of cortical apposition loss in the plane of maximal displacement.

Initial displacement severity was categorized as complete (no cortical contact) or incomplete (partial cortical contact preserved) and included as a covariate in multivariable analyses. Although angulation and translation were assessed, continuous measurements were not available as standardized, complete values for all patients in this retrospective cohort, and projection variability could introduce additional measurement uncertainty. Therefore, baseline displacement was modeled using a clinically interpretable categorical classification (cortical contact: complete vs. incomplete) to ensure consistent, reproducible classification across the dataset.

### 2.5. Treatment Pathways and Follow-Up Schedule

Primary conservative management consisted of closed reduction when needed and immobilization in a long-arm cast. Immediate post-reduction radiographs were obtained to document alignment.

Patients were followed using a standardized radiographic protocol during early healing with routine assessments at approximately 1, 2, 4, and 6 weeks after reduction.

For the purpose of outcome definition, the early follow-up period was predefined as the interval from index reduction to the 4-week assessment, as most clinically relevant secondary displacement occurs during this interval before early callus consolidation.

Primary surgical management was defined as operative fixation performed at index presentation without an initial trial of cast immobilization. Indications followed routine clinical practice and typically included inability to achieve acceptable alignment after reduction attempts, clearly unstable fracture configurations, or surgeon judgment documented in the medical record. When primary surgical fixation was selected, operative management was performed using standard pediatric forearm fracture techniques, including plate fixation or titanium elastic intramedullary nailing, in accordance with routine institutional practice and established principles described in the literature [10]. Primary surgical cases were included in descriptive analyses of management decisions but excluded from the loss-of-reduction risk model.

### 2.6. Acceptable Alignment Thresholds and Outcome Definitions

Acceptable alignment thresholds were predefined using age- and fracture-level-specific criteria derived from established pediatric remodeling principles and applied consistently at each follow-up assessment [11,12]. For mid-diaphyseal fractures, acceptable angulation was defined as 15 degrees or less for children younger than 10 years and 10 degrees or less for children 10 years or older, with translation not exceeding 50 percent.

For distal-third fractures, greater angulation was considered acceptable in younger children due to higher remodeling capacity, whereas proximal-third fractures were evaluated using stricter angulation thresholds given their comparatively limited remodeling potential. These level-specific criteria were applied systematically based on the dominant fracture level defined at baseline.

These predefined thresholds were used to determine whether alignment remained within acceptable limits during follow-up and to guide decisions regarding continuation of conservative treatment.

The primary outcome was early loss of reduction among patients managed with primary conservative treatment.

Early loss of reduction was defined as secondary displacement exceeding the predefined age- and level-specific acceptable alignment thresholds within the first four weeks after index reduction, following documentation of satisfactory immediate post-reduction alignment.

Only patients who achieved acceptable alignment on immediate post-reduction radiographs were eligible for subsequent classification as loss of reduction.

Loss of reduction required radiographic threshold violation and was considered clinically significant if it prompted re-manipulation, cast modification, or conversion to surgical fixation.

Secondary outcomes included the association between weight status and initial management decisions, fracture morphology, and conversion to surgery among conservatively managed patients.

Time to radiographic union was defined as the first follow-up visit at which bridging callus formation or cortical continuity was observed on orthogonal radiographs, accompanied by a clinical decision to discontinue immobilization in routine practice.

### 2.7. Statistical Analysis

All continuous variables analyzed in this study (age and follow-up duration) were assessed for normality using the Shapiro–Wilk test and showed no significant deviation from normality. Accordingly, these variables were summarized as mean (standard deviation). Categorical variables were summarized as frequencies and percentages. Between-group comparisons for continuous variables were performed using the independent-samples *t*-test, and categorical variables were compared using the chi-square test or Fisher’s exact test, as appropriate.

To evaluate independent predictors of early loss of reduction among conservatively treated patients, multivariable logistic regression was performed exclusively within the primary conservative cohort. The final model included obesity status, age, fracture level, and baseline displacement severity, selected a priori based on clinical relevance and constrained by the number of outcome events. Continuous radiographic measures (e.g., angulation and translation) were not entered into the multivariable model because standardized continuous measurements were not available for all patients; therefore, baseline displacement was modeled using a clinically interpretable categorical classification (complete vs. incomplete cortical contact).

Given the number of outcome events, the number of predictors was restricted to reduce overfitting, and covariate selection was guided by clinical plausibility rather than automated stepwise procedures. Adjusted odds ratios with 95% confidence intervals were reported. Model fit and potential multicollinearity were evaluated prior to final model acceptance. Statistical significance was defined as a two-sided *p*-value < 0.05. Multivariable analyses were performed using complete cases. All statistical analyses were conducted using IBM SPSS Statistics version 25.0.

## 3. Results

### 3.1. Patient Characteristics and Baseline Comparability

After application of the predefined clinical exclusion criteria, 895 children aged 3 to 13 years with acute both-bone forearm fractures were included in the final study cohort. Patients were stratified into normal-weight (*n* = 633, 70.7%) and obese (*n* = 262, 29.3%) groups according to age- and sex-adjusted BMI percentiles.

Baseline demographic and injury-related characteristics were generally similar between groups. Mean age did not differ significantly between the normal-weight and obese groups (7.84 (3.00) years vs. 7.73 (2.84) years, *p* = 0.715). Sex distribution, follow-up duration, and injury mechanism were also similar across weight categories (Table 1).

### 3.2. Fracture Characteristics According to Weight Status

Fracture morphology differed significantly between groups. Obese children presented with a substantially higher proportion of complete fractures compared to normal-weight children (82.4% vs. 61.9%, *p* < 0.001). Conversely, greenstick fractures were more common among normal-weight patients (38.1% vs. 17.6%) (Table 2).

In contrast, anatomical fracture level distribution did not differ significantly between groups (*p* = 0.301). The majority of fractures in both groups occurred in the distal third of the forearm (overall 77.7%), followed by mid-diaphyseal and proximal third fractures (Table 2).

### 3.3. Primary Treatment Selection at Index Presentation

The initial management strategy at presentation differed significantly by weight status (Table 3). Among normal-weight children, 13.1% underwent immediate operative fixation compared with 33.6% of obese children (*p* < 0.001). Accordingly, nonoperative management with closed reduction and casting was more frequently selected in normal-weight patients (86.9%) than in obese patients (66.4%).

Among those treated operatively, plate fixation was the most commonly used technique overall. Titanium elastic nailing was performed in 13.0% of obese patients and 2.8% of normal-weight patients (Table 3).

### 3.4. Early Loss of Reduction in the Primary Conservative Cohort

Of the 895 patients, 724 were initially managed with closed reduction and casting (normal weight *n* = 550; obese *n* = 174). Analyses of early loss of reduction were performed within this primary conservative cohort (*N* = 724).

Early loss of reduction occurred in 36 patients (5.0%). Loss of reduction was observed in 23 of 174 obese patients (13.2%) and in 13 of 550 normal-weight patients (2.4%), corresponding to an absolute risk difference of 10.8 percentage points (*p* < 0.001).

Refracture occurred in 3 obese patients (1.7%) and 3 normal-weight patients (0.5%) (*p* = 0.142).

Among the 66 overweight children (BMI 85th–94th percentile) excluded from the primary analysis to maintain a clearer two-group contrast, early loss of reduction occurred in 4 cases (6.1%). This rate was intermediate between the normal-weight (2.4%) and obese (13.2%) groups, suggesting a possible graded association across weight categories. The prespecified primary analysis, however, focused on the binary comparison (normal weight vs. obesity) to minimize heterogeneity around BMI cut-offs and maintain analytic consistency.

All cases classified as loss of reduction had documented acceptable immediate post-reduction alignment and subsequently exceeded predefined age- and fracture-level-specific alignment thresholds during the early follow-up period, and all prompted a change in management (re-manipulation and recasting/cast modification and/or conversion to surgical fixation).

### 3.5. Multivariable Analysis of Predictors of Early Loss of Reduction

To evaluate independent predictors of early mechanical failure, multivariable logistic regression was performed exclusively within the primary conservative cohort (*N* = 724). The final model included obesity status, age, fracture level, and baseline displacement severity, selected a priori based on clinical relevance and constrained by the number of outcome events.

In the adjusted model, obesity remained the strongest independent predictor of early loss of reduction (adjusted OR 5.98; 95% CI 2.89–12.38; *p* < 0.001) (Table 4). Complete displacement at presentation was not statistically significant (adjusted OR 1.94; 95% CI 0.77–4.90; *p* = 0.160).

## 4. Discussion

In this retrospective cohort study of children aged 3 to 13 years with both-bone forearm fractures, obesity was strongly associated with early loss of reduction after closed reduction and casting. In a multivariable model restricted to the primary conservative cohort, obesity remained independently associated with loss of reduction. Similar associations between higher BMI and failure of nonoperative management have been reported in pediatric both-bone forearm fractures, supporting the clinical relevance of weight status when counseling families and planning early follow-up [8].

Recent observational studies have also suggested that excess body mass may influence fracture characteristics, treatment selection, and short-term stability in pediatric upper-extremity injuries, further emphasizing the importance of considering weight status when evaluating fracture risk and treatment outcomes in children [13,14,15].

A key methodological feature of the present study was the separation of treatment selection from post-reduction mechanical failure. Obese children were more likely to undergo primary operative management at index presentation, which reflects clinical decision-making rather than cast stability. To avoid mixing these distinct endpoints, the risk analysis was limited to patients managed conservatively. This distinction is important because prior literature has often combined treatment choice and subsequent loss of reduction within a single denominator, which can obscure the true risk of mechanical failure among cast-treated children [2,16]. By explicitly restricting the multivariable model to the conservative cohort, the present study aimed to isolate mechanical failure after an initially acceptable reduction, thereby reducing potential bias related to surgeon decision-making and treatment selection. This analytical approach helps clarify whether obesity is associated with fracture instability during immobilization rather than merely reflecting differences in treatment preference.

The observed association between obesity and early loss of reduction is biologically and clinically plausible in the context of cast mechanics; however, causal inference is limited by the retrospective design and the absence of objective treatment-quality metrics. Established principles of three-point fixation and cast molding quality are important determinants of post-reduction stability, and quantitative cast indices have demonstrated value in predicting redisplacement in pediatric forearm fractures [17,18,19]. practical interpretation is that obesity may make cast immobilization less forgiving, because a larger soft-tissue envelope can reduce effective cast–limb control and compromise three-point fixation even when alignment meets acceptable thresholds immediately after reduction. This explanation remains hypothetical in our dataset because we did not measure cast quality indices, but it is consistent with the broader literature on cast mechanics and with studies examining cast-based predictors of redisplacement [5,17]. Accordingly, obesity should be interpreted primarily as a surrogate clinical risk marker associated with early loss of reduction rather than as a direct mechanical cause, and residual confounding due to unmeasured reduction quality, cast molding technique, and clinician-level factors cannot be excluded. Other investigators have similarly emphasized that cast technique, fracture configuration, and reduction quality remain dominant determinants of stability, and that patient-related factors such as obesity likely interact with these variables rather than acting as isolated causal mechanisms [20,21].

We also observed a substantially higher proportion of complete displacement in obese children, a morphology that is widely recognized as a risk factor for redisplacement across pediatric upper extremity fractures. Evidence syntheses and large cohorts of pediatric distal radius fractures consistently identify complete displacement and suboptimal reduction quality as major predictors of redisplacement after casting [22,23,24]. However, obesity remained associated with loss of reduction after adjustment for displacement severity and other covariates, suggesting that obesity’s effect is not fully explained by initial displacement alone and may reflect additional patient-related mechanical factors influencing cast stability [12]. Previous studies examining pediatric forearm fractures have similarly reported that higher BMI may be associated with more unstable fracture patterns at presentation, including complete cortical disruption or greater displacement, which may partially contribute to higher rates of secondary displacement during follow-up [25,26].

From a clinical perspective, the findings of the present study may have practical implications for follow-up strategies during the early post-reduction period. Most redisplacement events in pediatric forearm fractures occur within the first two to three weeks after reduction, before early callus formation provides additional mechanical stability [17]. In this context, children with obesity may benefit from careful cast molding and closer radiographic surveillance during early follow-up, particularly when fracture patterns are borderline stable [27]. Importantly, the results of the present study should not be interpreted as suggesting that obesity alone warrants primary surgical fixation. Rather, weight status should be considered as one of several clinical factors, alongside fracture morphology, reduction quality, and cast technique, when determining the intensity of early follow-up and counseling families regarding the potential risk of secondary displacement.

### Limitations

This study has limitations typical of a retrospective, single-center design. Treatment selection and follow-up intensity were determined in routine practice and may have varied across clinicians and over time, introducing potential selection bias and residual confounding, particularly in the analysis of initial operative management. Although the multivariable model was restricted to the primary conservative cohort and adjusted for clinically relevant covariates, unmeasured factors such as reduction quality and cast-molding indices were not routinely available. Therefore, obesity should be interpreted as a clinical risk marker associated with early loss of reduction rather than as a directly measured mechanical cause. In addition, the relatively small number of outcome events may have limited the precision of the multivariable effect estimate, which likely contributed to the relatively wide confidence interval around the adjusted odds ratio for obesity and warrants caution when interpreting its exact magnitude. Radiographic assessments were performed by two orthopaedic surgeons with consensus resolution of discrepancies, but formal interobserver reliability statistics were not calculated. Finally, outcomes were limited to early radiographic stability and treatment course, and the exclusion of overweight children, while methodologically useful for group separation, may reduce generalizability.

## 5. Conclusions

In this single-center cohort of children aged 3 to 13 years with acute both-bone forearm fractures, obesity was independently associated with a higher likelihood of early loss of reduction during nonoperative management after adjustment for clinically relevant covariates. Obese children also had a higher prevalence of complete displacement and were more likely to undergo primary operative management at index presentation. These findings support using weight status as a practical risk marker when counseling families and planning early follow-up for children treated with closed reduction and casting. Obesity alone should not be considered an indication for surgery, but it may justify a risk-adapted follow-up strategy that emphasizes meticulous cast molding and early radiographic reassessment, particularly during the first two weeks after reduction when loss of alignment most commonly occurs.

## Figures and Tables

**Table 1:** Baseline characteristics of the study cohort stratified by weight status (*N* = 895).

Variable	Normal Weight (*n* = 633)	Obese (*n* = 262)	*p*-Value
Age, years	7.84 (3.00)	7.73 (2.84)	0.715
Male sex, *n* (%)	485 (76.6)	192 (73.3)	0.331
Follow-up duration, weeks	9.42 (2.35)	9.50 (2.29)	0.629
Low-energy mechanism, *n* (%)	523 (82.6)	208 (79.4)	0.553

Values are presented as mean (SD) or number (%), as appropriate. Continuous variables were compared using the independent-samples *t*-test. Categorical variables were compared using the chi-square test or Fisher’s exact test, as appropriate. Statistical significance was defined as *p* < 0.05.

**Table 2:** Fracture morphology and anatomical level according to weight status (*N* = 895).

Variable	Normal Weight (*n* = 633)	Obese (*n* = 262)	*p*-Value
**Fracture type,** ***n*** **(%)**			<0.001
Complete	392 (61.9)	216 (82.4)	
Greenstick	241 (38.1)	46 (17.6)	
**Fracture level,** ***n*** **(%)**			0.301
Distal third	497 (78.5)	198 (75.6)	
Mid-diaphyseal third	123 (19.4)	61 (23.3)	
Proximal third	13 (2.1)	3 (1.1)	

Values are presented as number (%). Categorical variables were compared using the chi-square test or Fisher’s exact test, as appropriate. Statistical significance was defined as *p* < 0.05.

**Table 3:** Primary treatment selection according to weight status (*N* = 895).

Initial Management	Normal Weight (*n* = 633)	Obese (*n* = 262)	*p*-Value
Nonoperative (cast), *n* (%)	550 (86.9)	174 (66.4)	<0.001
Operative fixation, *n* (%)	83 (13.1)	88 (33.6)	
Plate fixation, *n* (%)	65 (10.3)	54 (20.6)	
Elastic intramedullary nail (TEN), *n* (%)	18 (2.8)	34 (13.0)	

Values are presented as number (%). The *p*-value reflects the comparison of the overall distribution of initial management between groups using the chi-square test. Subtypes of operative fixation are presented descriptively. Statistical significance was defined as *p* < 0.05.

**Table 4:** Multivariable logistic regression analysis for early loss of reduction in the primary conservative cohort (*N* = 724).

Predictor	Adjusted OR (95% CI)	*p*-Value
Obesity (vs. normal weight)	5.98 (2.89–12.38)	<0.001
Age (per year increase)	1.11 (0.98–1.25)	0.116
Complete displacement (vs. incomplete displacement)	1.94 (0.77–4.90)	0.160
Mid-diaphyseal level (vs. distal third)	0.61 (0.24–1.54)	0.295

Multivariable logistic regression was performed among patients managed with primary conservative treatment. The dependent variable was the early loss of reduction. The final model included obesity status, age, fracture level, and baseline displacement severity, selected a priori based on clinical relevance and constrained by the number of outcome events. Reference categories were normal weight, incomplete displacement, and distal third fracture level. Adjusted odds ratios with 95% confidence intervals are reported. Statistical significance was defined as *p* < 0.05.

## Data Availability

The data presented in this study are available on reasonable request from the corresponding author. The data are not publicly available due to institutional privacy regulations.

## Abbreviations

The following abbreviations are used in this manuscript:

BMI	Body mass index
CDC	Centers for Disease Control and Prevention
CI	Confidence interval
ICD-10	International Classification of Diseases, 10th Revision
OR	Odds ratio
aOR	Adjusted odds ratio
TEN	Titanium elastic nail

## References

[1] Landin L.A. (1983). Fracture Patterns in Children. Analysis of 8682 Fractures with Special Reference to Incidence, Etiology and Secular Changes in a Swedish Urban Population 1950–1979. Acta Orthop. Scand. Suppl..

[2] Sharma O., Hamidi D., Bozzo I., Alrajhi K., Bernstein M. (2025). Surgical and Conservative Management Are Both Effective for Pediatric Both Bone Forearm Fractures: A Systematic Review and Meta-Analysis. J. Pediatr. Orthop. Soc. N. Am..

[3] Li T.P., Wollstein A., Sabharwal S., Nayar S.K., Sabharwal S. (2022). Malunion of Pediatric Forearm Shaft Fractures: Management Principles and Techniques. Curr. Rev. Musculoskelet. Med..

[4] Šimunović I., Mrčela D., Karin Ž., Pogorelić Z., Markić J. (2024). Prevalence of Overweight and Obesity among Primary School Students in Split, Croatia. Nutrients.

[5] Alagöz E. (2020). Factors Affecting Re-Displacement in Pediatric Forearm Fractures and the Role of Cast Indices. Jt. Dis. Relat. Surg..

[6] McGurk K.M., Samaddar T., Barfield W.R., Murphy R.F. (2020). The Role of Obesity on Cast Index and Secondary Intervention in Pediatric Forearm Fractures. J. Orthop. Sci. Res..

[7] DeFrancesco C.J., Rogers B.H., Shah A.S. (2018). Obesity Increases Risk of Loss of Reduction After Casting for Diaphyseal Fractures of the Radius and Ulna in Children: An Observational Cohort Study. J. Orthop. Trauma..

[8] Lyons M., McGregor P.C., Hoyt A., Wozniak A., Cappello T., Fishman F.G. (2022). Do Forearm Fracture Characteristics and Outcomes Differ Between Obese and Non-Obese Children?. J. Pediatr. Orthop. Soc. N. Am..

[9] Ogden C.L., Kuczmarski R.J., Flegal K.M., Mei Z., Guo S., Wei R., Grummer-Strawn L.M., Curtin L.R., Roche A.F., Johnson C.L. (2002). Centers for Disease Control and Prevention 2000 Growth Charts for the United States: Improvements to the 1977 National Center for Health Statistics Version. Pediatrics.

[10] Pogorelić Z., Gulin M., Jukić M., Biliškov A.N., Furlan D. (2020). Elastic Stable Intramedullary Nailing for Treatment of Pediatric Forearm Fractures: A 15-Year Single Centre Retrospective Study of 173 Cases. Acta Orthop. Traumatol. Turc..

[11] Noonan K.J., Price C.T. (1998). Forearm and Distal Radius Fractures in Children. J. Am. Acad. Orthop. Surg..

[12] Scotcher M., Chong H.H., Asif A., Kulkarni K. (2023). Radiological Criteria for Acceptable Alignment in Paediatric Mid-Shaft Forearm Fractures: A Systematic Review. Malays. Orthop. J..

[13] Taylor E.D., Theim K.R., Mirch M.C., Ghorbani S., Tanofsky-Kraff M., Adler-Wailes D.C., Brady S., Reynolds J.C., Calis K.A., Yanovski J.A. (2006). Orthopedic Complications of Overweight in Children and Adolescents. Pediatrics.

[14] Nowicki P., Kemppainen J., Maskill L., Cassidy J. (2019). The Role of Obesity in Pediatric Orthopedics. J. Am. Acad. Orthop. Surg. Glob. Res. Rev..

[15] Yang W., Zhou Z., Gu W., Wang X., Ni L. (2025). The Impact of Childhood Obesity on Different Fracture Sites. Sci. Rep..

[16] Caruso G., Caldari E., Sturla F.D., Caldaria A., Re D.L., Pagetti P., Palummieri F., Massari L. (2021). Management of Pediatric Forearm Fractures: What Is the Best Therapeutic Choice? A Narrative Review of the Literature. Musculoskelet. Surg..

[17] Alemdaroğlu K.B., İltar S., Çimen O., Uysal M., Alagöz E., Atlıhan D. (2008). Risk Factors in Redisplacement of Distal Radial Fractures in Children. J. Bone Jt. Surg..

[18] Asadollahi S., Ooi K.S., Hau R.C. (2015). Distal Radial Fractures in Children: Risk Factors for Redisplacement Following Closed Reduction. J. Pediatr. Orthop..

[19] Franklin C.C., Robinson J., Noonan K., Flynn J.M. (2012). Evidence-Based Medicine: Management of Pediatric Forearm Fractures. J. Pediatr. Orthop..

[20] Chess D.G., Hyndman J.C., Leahey J.L., Brown D.C.S., Sinclair A.M. (1994). Short Arm Plaster Cast for Distal Pediatric Forearm Fractures. J. Pediatr. Orthop..

[21] LaValva S.M., Rogers B.H., Arkader A., Shah A.S. (2020). Risk Factors for Failed Closed Reduction of Pediatric Distal Radius Fractures. J. Hand Surg. Glob. Online.

[22] Sengab A., Krijnen P., Schipper I.B. (2020). Risk Factors for Fracture Redisplacement after Reduction and Cast Immobilization of Displaced Distal Radius Fractures in Children: A Meta-Analysis. Eur. J. Trauma. Emerg. Surg..

[23] Shah A.S., Belardo Z.E., Miller M.L., Willey M.C., Mahan S.T., Talwar D., Bae D.S. (2025). Loss of Reduction in Pediatric Distal Radius Fractures: Risk Factors From a Prospective Multicenter Registry. J. Pediatr. Orthop. Soc. N. Am..

[24] Bae D.S. (2008). Pediatric Distal Radius and Forearm Fractures. J. Hand Surg..

[25] Ryan L.M., Teach S.J., Searcy K., Singer S.A., Wood R., Wright J.L., Hunting K.L., Chamberlain J.M. (2015). The Association Between Weight Status and Pediatric Forearm Fractures Resulting From Ground-Level Falls. Pediatr. Emerg. Care.

[26] Kohan Fortuna Figueira S.V., Saralegui P., Magno G.M., Bosio S.T., Slullitel P., Rossi L., Camino-Willhuber G. (2024). Pediatric Fracture of the Forearm and Wrist. Orthopaedics and Trauma.

[27] Vescio A., Testa G., Sapienza M., Caldaci A., Montemagno M., Andreacchio A., Canavese F., Pavone V. (2022). Is Obesity a Risk Factor for Loss of Reduction in Children with Distal Radius Fractures Treated Conservatively?. Children.

